# Large spiking AI systems

**DOI:** 10.1093/nsr/nwag104

**Published:** 2026-02-12

**Authors:** Steve Furber

**Affiliations:** Emeritus professor at the University of Manchester, UK

## Abstract

A recent work on a spike-based LLM demonstrates the feasibility and desirability of applying biological principles to address the current `deathly embrace' between AI algorithms and GPU-based compute platforms.

Recent work on a spike-based large language model [1] demonstrates the feasibility and desirability of applying biological principles to address the current ‘deathly embrace’ between AI algorithms and GPU-based compute platforms, which is responsible for the unsustainable energy requirements of today’s large-scale AI systems.

Advances in large language models have brought spectacular results, but at very high energy costs. These costs are incurred in training, where the tens or hundreds of billions of parameters in a network must be repeatedly adjusted by exposure to vast data sets until optimal performance is achieved, and in the inference phase, where those parameters are subject to repeated dense matrix operations—operations at which GPU-based systems excel. In contrast, biological systems can learn from modest data sets, and their intrinsic matrix operations are always sparse, both in space and in time.

Why, then, do large AI systems ignore the obvious efficiency benefits of spatial and temporal sparsity? There are several possible answers. First, GPUs are the established compute platform for AI, and GPUs do not favour sparse matrix operations: they are designed to compute everything, all of the time. Second, although there has been progress in sparsifying neural networks during and after training, this remains an under-explored area. Third, although the human brain (and other animal brains) provides an existence proof that sparse neural networks can be highly energy efficient, we do not know enough about the brain to be able to use brain-like neural networks extensively in engineered AI systems.

This paper [1] represents a significant contribution to advancing the field of more efficient, more brain-like large-scale AI. The authors also point out that their approach contributes to interpretability—that is, progress towards explainable AI—which is visibly lacking in current mainstream AI systems. There is also the prospect that such work will lead to advances in brain science, towards an understanding of the fundamental principles underlying computation in the brain.

The work uses an FPGA as the hardware platform, which raises the question of how the results might differ if they were based on a large-scale neuromorphic computing platform—such systems are already available [2]. It is possible that this question will be addressed in follow-on work in the near future.

Overall the paper advances the case for the convergence of AI with neuromorphic computing, for greater use of biological principles in AI, and for the development of event-driven computational models supporting considerable degrees of both spatial and temporal sparsity in AI systems, resulting in significant improvements in the energy-efficiency of such systems.

The paper also speculates on the potential for such systems to contribute to the development of AGI, while recognising that this is not only a technical but also a philosophical challenge, raising significant ethical questions about the societal impact of AGI.

***Conflict of interest statement.*** None declared.
